# p21 is decreased in polycystic kidney disease and leads to increased epithelial cell cycle progression: roscovitine augments p21 levels

**DOI:** 10.1186/1471-2369-8-12

**Published:** 2007-08-22

**Authors:** Jin-Young Park, William E Schutzer, Jessie N Lindsley, Susan P Bagby, Terry T Oyama, Sharon Anderson, Robert H Weiss

**Affiliations:** 1 Immunology Graduate Group, University of California, Davis, CA, USA; 2 Division of Nephrology, Dept. of Internal Medicine, University of California, Davis, CA, USA; 3 Division of Nephrology and Hypertension, Dept. of Medicine, Oregon Health and Science University, Portland, OR, USA; 4 Research Service, Portland VA Medical Center, Portland, OR, USA; 5 Medical Service, Sacramento VA Medical Center, Sacramento, CA, USA

## Abstract

**Background:**

Autosomal dominant polycystic kidney disease (ADPKD) is a common genetic disease with few treatment options other than renal replacement therapy. p21, a cyclin kinase inhibitor which has pleiotropic effects on the cell cycle, in many cases acts to suppress cell cycle progression and to prevent apoptosis. Because defects in cell cycle arrest and apoptosis of renal tubular epithelial cells occur in PKD, and in light of earlier reports that polycystin-1 upregulates p21 and that the cyclin-dependent kinase inhibitor roscovitine arrests progression in a mouse model, we asked whether (1) p21 deficiency might underlie ADPKD and (2) the mechanism of the salutary roscovitine effect on PKD involves p21.

**Methods:**

p21 levels in human and animal tissue samples as well as cell lines were examined by immunoblotting and/or immunohistochemisty. Apoptosis was assessed by PARP cleavage. p21 expression was attenuated in a renal tubular epithelial cell line by antisense methods, and proliferation in response to p21 attenuation and to roscovitine was assessed by the MTT assay.

**Results:**

We show that p21 is decreased in human as well as a non-transgenic rat model of ADPKD. In addition, hepatocyte growth factor, which induces transition from a cystic to a tubular phenotype, increases p21 levels. Furthermore, attenuation of p21 results in augmentation of cell cycle transit *in vitro*. Thus, levels of p21 are inversely correlated with renal tubular epithelial cell proliferation. Roscovitine, which has been shown to arrest progression in a murine model of PKD, increases p21 levels and decreases renal tubular epithelial cell proliferation, with no affect on apoptosis.

**Conclusion:**

The novelty of our study is the demonstration *in vivo *in humans and rat models of a decrement of p21 in cystic kidneys as compared to non-cystic kidneys. Validation of a potential pathogenetic model of increased cyst formation due to enhanced epithelial proliferation and apoptosis mediated by p21 suggests a mechanism for the salutary effect of roscovitine in ADPKD and supports further investigation of p21 as a target for future therapy.

## Background

Autosomal dominant polycystic kidney disease (ADPKD) is a common genetic disorder and, due to the absence of any currently available effective treatment, the most common genetic cause of end-stage renal disease (ESRD). While the pathogenesis of ADPKD is often viewed in light of defects in trans-epithelial fluid transport and associated channels causing massive and abundant cyst formation, it is becoming increasingly clear that ADPKD is also a disease of disordered epithelial cell cycle regulation and apoptosis. In addition, due to its monoclonality in relation to proliferating renal tubular epithelial (RTE) cells, ADPKD has been described as "a neoplasm in disguise" [1]. In kidneys from ADPKD patients, cystic epithelial cells have a markedly high mitotic rate as measured by a variety of proliferative markers, and an increased level of apoptosis[2], findings which may be due to the failure of appropriate cell cycle inhibition. Thus, a somewhat simplistic view of ADPKD, from a cell cycle perspective, is one of increasing tubular epithelial proliferation and failure of normal apoptotic inhibition.

The cyclin-dependent kinase inhibitors (CKI) function in mitogenic signaling through their interaction with the cyclins and the cyclin dependent kinases [3], and through proliferating cell nuclear antigen (PCNA) [4]. The CKIs influence apoptosis through interaction with a variety of second messengers [5], and the CKI p21 specifically has been shown to possess pleiotropic effects on cell division and apoptosis in a variety of cell systems, such that the net result of p21 activation is variable and dependent on cell type, as well as on its subcellular localization [5-8]. Due to the pivotal role of epithelial cell cycle regulation in the generation of PKD cysts, and in light of the similarities of PKD to neoplasia, examination of p21 is a logical potential target for novel therapeutic approaches in this disease.

Germino et al[9] were the first to suggest a relationship between p21 and PKD by showing that p21 is induced by the product of the *Pkd1 *gene, polycystin-1. While these investigators also showed that p21 levels were reduced in *Pkd1 *knockout mice, there has to our knowledge been no published study examining the function of p21 in human tissues or in non-knockout animal models of this disease. In this study, we examine the expression of p21 in human tissue as well as in rat tissues. Very recent data has supported the role of cyclin kinase inhibition as possible treatment of PKD, using the specific inhibitor roscovitine[10].

We now show that p21 is consistently downregulated in all models examined. Furthermore, in the commonly used Han:SPRD rat model, where the mutation is in the *Pkdr1 *gene [11], there appears to be a male gender-specific predilection for p21 attenuation, which is consistent with observed gender dimorphism of disease severity in clinical and experimental studies [12,13]. We have also begun to evaluate whether p21 is pathogenic in ADPKD, by showing that hepatocyte growth factor (HGF), which induces transition from a cystic to a tubular phenotype *in vitro*, increases p21 levels, and that levels of p21 correlate inversely with proliferation of RTE cells. Finally, we show that not only does roscovitine decrease proliferation of RTE cells, but it also increases p21 and consequently does not cause increased apoptosis at low doses, suggesting a mechanism for its salutary effect in this disease.

## Methods

### Experimental models

Han:SPRD rats: These studies were conducted in Han:SPRD rats, from a colony propagated from breeding pairs kindly provided by Dr. Benjamin D. Cowley, Jr. (then at the University of Kansas Medical Center, Kansas City, KS). Male and female heterozygous cystic rats (Cy/+) and unaffected littermate control rats (+/+) were raised to 12 weeks of age. After anesthesia with Inactin (100 mg/kg i.p.), kidneys were removed and frozen at -80° for protein expression studies, and put into 10% formalin for immunohistochemistry (IHC) studies as below. The Portland Veterans Affairs Institutional Animal Care and Use Subcommittee approved these studies.

Human ADPKD: End stage kidneys from 11 male patients with ADPKD and kidney samples from 5 male patients without ADPKD (without fibrosis) were obtained at surgical nephrectomy via institutional review board (IRB)-approved protocols (OHSU IRB 6044VAW; PVAMC IRB 00349). After nephrectomy, ADPKD kidneys were stored on ice during transport. For IHC, tissue was fixed in 10% formalin and processed for paraffin blocks for storage until section at 5 μm.

### Immunoblotting

The Han:SPRD Rat and human kidneys were homogenized in solubilization buffer (50 mM TRIS, 150 mM NaCl, 0.5% sodium deoxycholate, 0.1% SDS, 1.0% Triton-X 100, and protease inhibitors [leupeptin, 20 μg/ml; benzamidine, 20 μg/ml; phenyl methylsulfonyl fluoride [PMSF], 40 μg/ml)]). These homogenates were centrifuged at 12,000 × g for 30 min at 4°C and supernatants were collected. Total protein content was determined by BCA analysis (Pierce Chemical Co., Rockford, IL).

Denatured proteins were separated through a 12% SDS-polyacrylamide gel and transferred to PVDF membranes (Bio-Rad Laboratories, Hercules, CA). Membranes were washed and blocked overnight with TRIS buffered saline 0.05% Tween-20 (TBS-T), containing 5% nonfat dry milk. Following blocking, membranes were again washed (TBS-T), and incubated overnight with a rabbit polyclonal anti-human p-21 (H-164; Santa Cruz Biotechnology Inc., Santa Cruz, CA), diluted 1:3000 in TBS-T. Immunodetection was accomplished by incubating membranes with a goat anti-rabbit-IgG secondary antibodies conjugated with horseradish peroxidase (HRP) for 45 minutes (Pierce, 1:100,000) in TBS-T containing 5% nonfat dry milk. Visualization was performed with enhanced chemiluminiscence (ECL) western-blotting kit (Supersignal West Dura, Pierce) according to the manufacturer's instructions. The membranes were then stripped, re-blocked, and re-incubated for 1 hr at room temperature with goat Actin antibody (I-19; Santa Cruz), 1:4000, followed by 45-min incubation with anti-goat-IgG secondary antibody conjugated with HRP (1:40,000, Santa Cruz), and reaction with ECL. Resultant films (Eastman Kodak Co., Scientific Imaging Systems, New Haven, CT) were scanned using a flatbed scanner and image analysis was performed with the ImageJ gel analysis algorithm.

### Immunohistochemistry

The same antisera as described above were used for immunohistochemical detection of rat or human p21. Sections were deparaffinized in xylene, rehydrated through graded ethanols to water, and pretreated by steaming in 10% CITRA buffer (BioGenex Laboratories, San Ramon, CA). After being treated with protein-blocking solution, the slides were incubated overnight at 4°C with primary antibody (diluted 1:500) or with the same concentration of nonimmune IgG as a control. Endogenous peroxidase activity was blocked with 3% H_2_O_2 _solution in methanol. The primary antibody was localized using the Vectastain ABC-Elite peroxidase detection system (Vector Laboratories, Burlingame, CA). This was followed by reaction with diaminobenzidine as chromogen and counterstaining with hematoxylin (Sigma). Sections of each polycystic kidney were processed in parallel with appropriate control tissue. Visualization occurred with a Zeiss Axioskop microscope connected to a Micropublisher 3.3 digital camera (QImaging; Burnaby, B.C., Canada) interfaced with a Macintosh G5 computer (Apple, Cupertino, CA) utilizing QCapture software (QImaging).

### Digital image processing

After data acquisition, raw images were opened with Photoshop 7.0 for Macintosh (Adobe; San Jose; CA) and images were processed with the built-in adjustments function of "auto levels," "auto contrast," and "auto color." No further image manipulation occurred.

### Cell culture and antisense transfections

MDCK (Madin-Darby canine kidney) cells were obtained from Dr. John Payne at UC Davis. The cells were maintained in Dulbecco's media and 10% FBS and 1% Penicillin/Streptomycin.

The canine p21 antisense oligodeoxynucleotide (ODN; sequence 5'-GTC-AAA-GTT-CCA-TCG-CTC-CC-3') was synthesized by Molecula (Stering, VA). Control ODN (5'-GGA-CTA-CTA-CAC-TAG-ACT-AC-3', designed and tested by the vendor) was synthesized by Biognostik (Germany). For the transfection procedure, 1 × 10^5 ^cells were seeded in 6 well plates 24 hr before transfection and cells were washed with sterile PBS before the transfection. The transfection reagent, GeneJuice (Novagen, Germany) was diluted with OptiMEM first and then the ODNs were added in diluted GeneJuice mixture. The ODN mixture was added to the cells for 24 hr.

### MTT assay

MDCK cells were seeded in 96 well plate 24 h before treatment with ODN or roscovitine (Sigma). For ODN transfection, 200 nM and 400 nM antisense or control ODN were added into the cells and incubated for 24 h. For roscovitine treatment, 1–10 ug/ml roscovitine was added in serum-containing media and incubated for 24 h. Media containing ODNs or roscovitine was removed and cells were incubated with 50 μg/ml MTT (Sigma) for 4 h at 37°C. Purple formazan was solubilized and samples were measured by multiwell scanning spectrophotometer at 570 nm.

### Statistical analysis

Values are reported as means ± SEM. Statistical analysis was performed by unpaired t test, or by analysis of variance followed by computation of modified *t *values according to the method of Bonferroni (for multiple groups), as appropriate. Statistical significance was defined as p < 0.05. Values are means ± SEM.

## Results

### p21 is decreased in ADPKD patients and in rat models of PKD

**Figure 1:**
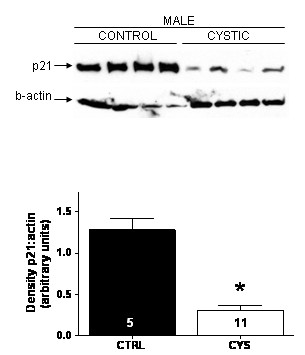
p21 is decreased in kidneys from ADPKD patients. [top] Kidney protein extracts from control (closed) and PKD (open) human males were immunoblotted with an anti-p21 and β-actin antibody. Immunoblots were analyzed by densitometry with ImageJ as described in *Methods*. [bottom] p21 expression is presented as the ratio of intensity of p21 bands compared to intensity of actin bands presented as mean ± SEM. * p < 0.01 versus within gender control. Values within each bar denote the number of kidneys utilized.

**Figure 2:**
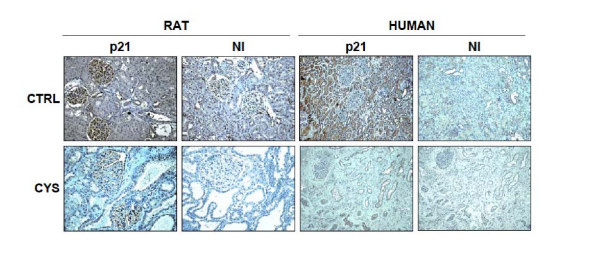
Representative immunohistochemical expression of p21 in male cystic and noncystic Han:SPRD rats (left), and male PKD and control humans (right). Upper panels show p21 localization from control sections, and the lower panels show cystic sections. Also shown are sections (NI) pre-incubated with non-immune serum that demonstrate antibody specificity. In sections from control male kidneys, p21 was widely distributed, whereas in sections from cystic male kidneys, p21 was only sparsely detected.

While p21 has been shown to be induced by polycystin-1[9], there are as yet no studies demonstrating that a lack of p21, as would be expected with mutant *PKD1*, occurs *in vivo *in humans or in those animal models not characterized by targeted gene disruption. We tested the hypothesis that p21 is downregulated in human ADPKD tissues, and then further tested generalizability of the findings in a rat non-knockout model. By immunoblotting, renal expression of p21 was significantly attenuated in cystic kidneys from human ADPKD patients, as compared with kidneys from noncystic control patients (Fig. 1; p < 0.05). By immunohistochemical analysis, there was intense p21 staining in normal kidneys. This staining was most pronounced in the renal tubules, with relatively sparse staining in glomeruli. In the cystic kidneys, staining was essentially absent in all kidney segments (Fig. 2, right panels), similar to slides incubated with non-immune serum (incubation with a normal rabbit IgG primary antibody; NI in Fig. 2).

There exist several animal models of ADPKD, each with its distinct advantages and disadvantages. Of the available rodent models, the Han:SPRD rat is commonly used, and may be the only rodent model which progresses to ESRD. We evaluated control and cystic kidneys from male Han:SPRD rats, and then sought to determine whether the observed changes were replicated in female rats. General characteristics of the Han:SPRD rats used are depicted in Table 1. Females were smaller than males, but the cystic phenotype did not affect body weight. The kidney and kidney:body weight ratios were markedly increased in cystic males, as compared with noncystic controls (p < 0.01). In cystic females, renal enlargement was also present, but to a significantly lesser magnitude than was seen in cystic males (p < 0.01). These results are consistent with prior observations of gender dimorphism with respect to progression of cystic disease in this model [13,14].

**Table 1:** General characteristics of Han:SPRD rats (12 weeks old)

	**Males**		**Females**	
	Control	Cystic	Control	Cystic

	(n = 9)	(n = 12)	(n = 7)	(n = 11)

Body wt (gms)	385 ± 8	356 ± 7	253 ± 4†	242 ± 4†
LKW (gms)	1.47 ± 0.05	4.19 ± 0.13*	0.86 ± 0.03†	1.89 ± 0.04*†
LKW/100 gms BW	0.38 ± 0.01	1.18 ± 0.03*	0.34 ± 0.01	0.76 ± 0.01*†

Values are means ± SEM. LKW, left kidney weight; BW, body weight. * p < 0.01 vs. respective noncystic control; † p < 0.01, female vs. respective male group

**Figure 3:**
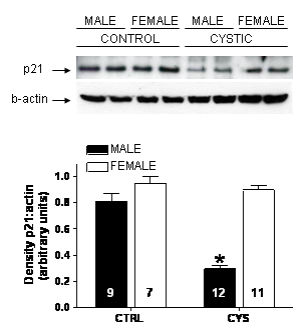
p21 is decreased in kidneys from male Han:SPRD rats. [top] Protein extracts in kidney cortical preparations from control (closed) and cystic (open) male and female Han:SPRD rats were immunoblotted with an anti-p21 and β-actin antibodies. Immunoblots were analyzed by densitometry with ImageJ as described in *Methods*. [bottom] p21 expression is presented as the ratio of intensity of p21 bands compared to intensity of actin bands presented as mean ± SEM. * p < 0.01 versus control of same gender. Values within each bar denote the number of animals utilized.

Expression of p21 in cortical homogenates from control and cystic Han:SPRD animals was assessed by immunoblotting (Fig. 3). Renal expression of p21 was significantly attenuated in cystic male rats, as compared with noncystic controls (p < 0.01). In contrast, renal p21 expression in cystic females did not differ from that seen in noncystic control females. Immunohistochemical expression studies mirrored the findings from immunoblotting. In noncystic control male rats, p21 staining was evident in the glomeruli as well as the tubulointerstitial compartment (Fig. 2, top left), which was somewhat different from the human tissues which showed relatively sparse glomerular staining. However, in cystic males, staining was much less intense, particularly in the tubulointerstitial and cystic compartments (Fig. 2, bottom left). Non-immune experiments (incubation with a normal rabbit IgG primary antibody) yielded little or no signal (NI in Fig. 2).

### HGF, which induces transition from a cystic to a tubular phenotype, induces p21

MDCK cells, a canine RTE cell line, have been utilized in *in vitro *PKD studies for many years, and thus the cystic behavior of these cells has been well characterized. MDCK cells spontaneously form cysts when cultured in 3D collagen gels and undergo *in vitro *tubulogenesis when treated with HGF; thus HGF functions as a "molecular switch" from cyst to tubule formation [15]. Interestingly, while not evaluated in relation to a cystic phenotype, p21 has been shown to be induced by HGF in several cell lines [16], consistent with our hypothesis that p21 mediates the tubulogenic, not the cystic, phenotype. Thus, these cells are ideal for examining the role of p21 on cyst formation.

**Figure 4:**
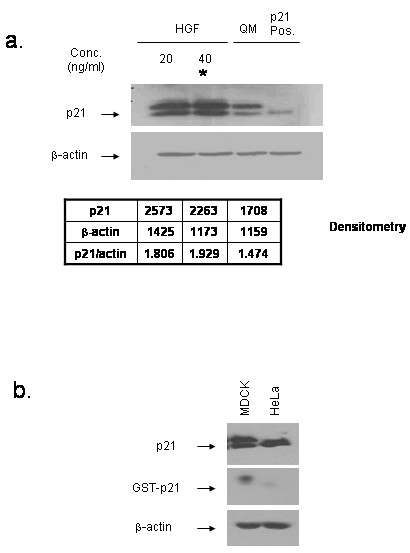
HGF increases p21 in MDCK cells. a. MDCK cells were seeded and serum starved for 24 h. HGF (20 and 40 ng/ml) was added for 24 h in serum-free quiescence media and the cells were lysed and immunoblotted with anti-p21 and β-actin antibodies. Human p21 was used for p21 positive control and indicates that lower band of doublet is p21. QM: quiescence media; p21 pos: p21 positive control (HeLa cell lysate). Densitometry is shown; *p < 0.05 as compared to QM alone. Experiment shown is representative of two experiments. b. In order to determine which band of the doublet represents p21, MDCK and HeLa control cell lysate were immunoblotted with p21 antibody alone or with p21 antibody which had been pre-incubated with GST- tagged p21 antigen, demonstrating that the lower molecular weight band is p21.

MDCK cells were serum-starved in order to synchronize them in G_o _phase and were subsequently incubated with recombinant human HGF at 20 and 40 ng/ml for 24 h, and immunoblotted with p21 antibody. HGF stimulation of these cells at 20 and 40 ng/ml resulted in the induction of p21 for 24 hours (Fig. 4a). Due to the doublet observed with this antibody in MDCK cell lysate, we utilized antigen competition in order to determine that the lower molecular weight band represents p21 (Fig. 4b). Thus, p21 is downstream of HGF and may mediate its function in maintaining the tubular (and hence non-cystic) phenotype [15], such that its absence contributes to cyst formation. While this data is consistent with our finding that p21 is decreased in cystic tissue, whether HGF lies within the polycystin 1 → p21 pathway remains under active investigation in our laboratories.

### Attenuation of p21 results in increased tubular epithelial cell proliferation

One of the functions of p21 is to arrest cell proliferation by virtue of its association with PCNA (thereby eliminating the action of PCNA on transcription) and/or by its inhibition of cyclin/cdk association [4,17,18]. However, it is now clear that this property of p21 is cell-type dependent, since p21 can also, under some contexts, function as a cyclinD/cdk4 assembly factor [19,20] and has variable effects on apoptosis [5,21,22]. We next asked whether attenuation of p21 in MDCK cells, which have the potential to form either cysts or tubules [23], results in enhanced cell cycle transit. In the context of its putative role in PKD as an inhibitor of cystogenesis, p21 would be expected to decrease RTE cell proliferation and thus prevent cyst formation.

**Figure 5:**
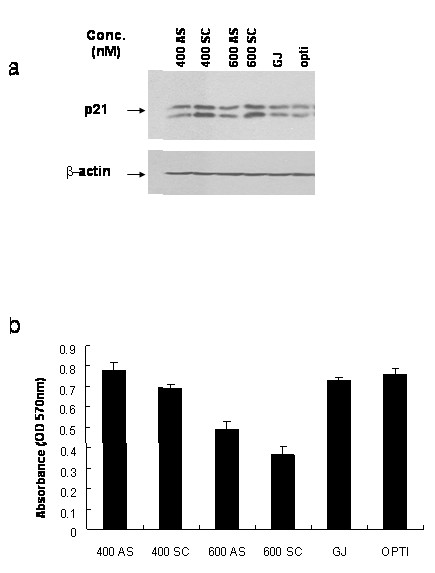
Levels of p21 are inversely proportional to MDCK cell proliferation. a. MDCK cells were seeded 24 h before transfection, and transfected with antisense p21 (AS) or scrambled sequence control (SC) ODN in serum-reduced medium as described in *Methods*. Cell lysates were immunoblotted with anti-p21 and β-actin antibodies. GJ: Gene-Juice (transfection reagent); opti: Optimem medium. b. MDCK cells transfected with antisense p21 or control ODNs were incubated with MTT and purple soluble formazan was assessed as described in *Methods*. Experiments shown are representative of at least two experiments.

We utilized an antisense ODN approach, which we have previously found to be successful in both human[21] and rat [20] cell lines. We have previously shown that a 21-mer antisense ODN to human p21 has a nearly 100% transfection efficiency in human cells [20]. We designed a 20-mer phosphorothioated antisense ODN using the canine p21 sequence and demonstrated that this construct, when transiently transfected into MDCK cells at both 400 and 600 nM, results in attenuation of p21 when compared to an equivalent concentration of scrambled control ODN (Fig. 5a). Specificity of the canine ODN was demonstrated by its lack of effect on actin protein levels.

Cell cycle progression of p21-attenuated MDCK cells was assessed by the MTT proliferation assay. Antisense p21 ODN transfection of MDCK cells at 400 and 600 nM showed increased cell proliferation as compared to cells transfected with scrambled control ODN (Fig. 5b). The increase in p21 with scrambled control ODN was likely a result of non-specific cellular stress and 600 nM ODNs are more toxic to cells than 400 nM, showing the overall lower proliferation rate; however, the levels of p21 (Fig. 5a) correspond inversely with cell proliferation (Fig. 5b), consistent with the established cell cycle inhibitory effect of p21, and supporting our hypothesis that decreased p21 as seen in PKD is associated with increased RTE proliferation. Thus, p21 is playing a growth suppressive role in RTE cells, such that the lower levels of p21 observed in cystic tissue is likely resulting in increased cell cycle transit and contributing to cyst formation.

### Roscovitine decreases cell proliferation in parallel with p21 augmentation

**Figure 6:**
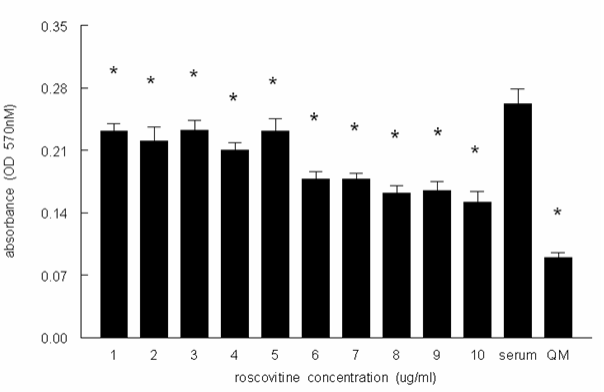
Roscovitine decreases proliferation and increases p21 with no effect on apoptosis. MDCK cells were seeded 24 h before treatment. Roscovitine (at the concentrations indicated) was added to the cells for 24 h and then the cells were harvested and subjected to the MTT assay as described in Materials and Methods. QM is serum-free media; * p < 0.05 compared to serum alone. Experiment shown is representative of at least three experiments.

The potent CDK inhibitor roscovitine has been recently shown to arrest PKD in a mouse model[10]. We asked whether the MDCK model of PKD that we have employed in this study responds as expected to this inhibitor. When incubated with a range of roscovitine concentrations, proliferation of MDCK cells, as measured by the MTT assay, showed a dose dependent decrease, reaching significance at all concentrations (Fig. 6).

**Figure 7:**
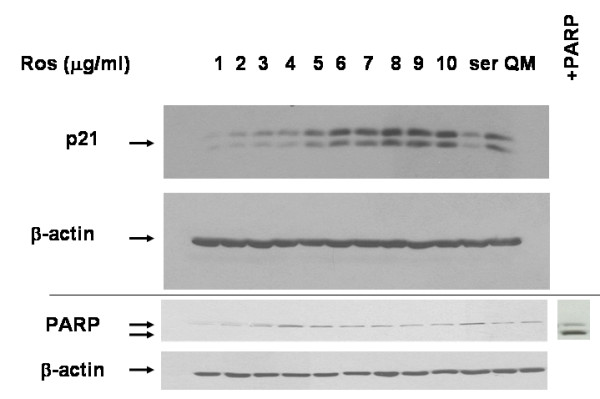
Roscovitine increases p21 with no effect on apoptosis. MDCK cells were seeded 24 h before treatment. Roscovitine (at the concentrations indicated) was added to the cells for 24 h and then the cells were harvested and subjected immunoblotted with p21, β-actin, and PARP antibodies. PARP control (+PARP) represents cells treated with the DNA damaging agent doxorubicin showing the lower molecular weight cleavage band. QM is serum-free media; ser is serum-containing media.

Consistent with our model of p21 decrease being associated with the cystic phenotype, we found that p21 was induced in a dose dependent fashion by roscovitine (Fig. 7), suggesting a mechanism for the roscovitine effect proximal to cyclin-cdk inhibition. Since PKD is associated with increase RTE apoptosis[2] and p21 possesses an anti-apoptotic function in several cell lines [5,21], we would expect potential drug therapies for PKD to not be associated with programmed cell death, in contrast to what would be expected in the cancer chemotherapy setting [24]. Thus, while we have in fact observed apoptosis at higher concentrations of roscovitine associated with decreased p21, consistent with its chemotherapeutic utility (data not shown), there was no increase in apoptosis, as measured by the absence of a PARP cleavage (lower molecular weight) band, in cells incubated with this p21-augmenting lower concentration of roscovitine (Fig. 7). In addition, the lack of apoptosis seen here supports the likelihood that the MTT assay reflects a decrease in cell proliferation rather than an increase in apoptosis.

## Discussion

ADPKD is one of the most common genetic diseases and is the most common hereditary renal disease in the US, accounting for 4.7% of ESRD population, and for which the cost of medical care exceeds $200 million per year. While no specific therapies currently exist for this disease, recent research into the pathogenesis of cyst formation, utilizing both animal models of the disease as well as cell culture systems which form cysts, has revealed pathways which will ultimately lead to the development of novel therapeutic approaches. One of these pathways involves key arbiters of cell cycle progression and apoptosis, the cyclins, their associated kinases, and the cyclin kinase inhibitors, of which group p21 is a member. The concept of targeting the cyclin pathway for PKD therapy has been reinforced recently with the publication of a study in a murine PKD model (*jck *mice) showing that the cancer chemotherapeutic agent and CDK inhibitor, roscovitine, has long-term beneficial effects on disease progression [10]. In fact, this study is consistent with the concept of cyst formation in ADPKD being a monoclonal proliferation of polycystin-deficient RTEs, the disease having similarities to neoplasia [1]. In addition, the observed increased rate of renal tissue apoptosis in PKD[2] further supports the investigation of cancer-relevant signaling proteins as potential targets for this disease.

While the cystic epithelium in ADPKD has well described derangements in ion transporters (such as the Na+/K+-ATPase), ADPKD is also a disease of both hyperactive RTE proliferation and increased apoptosis. Research into molecular pathways involved in cystogenesis has markedly accelerated since the elucidation of the genetic defects which accompany ADPKD. In particular, those signaling events which lead to increased proliferation of cystic epithelium are now being dissected out in several laboratories. In this study, we have built on the observation that polycystin-1, the gene product of *PKD1 *which is mutated in 85% of ADPKD cases, induces the cyclin kinase inhibitor p21[9]. We have shown for the first time that this paradigm holds up in humans as well as in an established rat model of this disease. In addition, our data is consistent with the known anti-apoptotic function of p21 elucidated in our and others' laboratories [5,21,25], since ADPKD is known to be associated with increased apoptotic loss of renal tissue[2], a finding which would be expected to occur in the presence of the reduced levels of p21 which we observed in this study.

The cyclin-dependent kinases (CDKs) are a well-conserved family of serine/threonine protein kinases (one in yeast and at least 8 in animals) which function in mitogenic signaling through their activation by the cyclins. This in turn leads to a cascade of events whereby the mitogen-stimulated, cyclin D-dependent kinases phosphorylate retinoblastoma (Rb) protein, causing release of phosphorylated Rb from the transcription factor family known as E2F, and allowing S-phase specific gene transcription and subsequent progression through the G_1_/S transition. Cell cycle progression in normal, non-transformed (e.g. epithelial) cells is finely regulated by the interplay between the cdks and the CKIs (such as p21 and p27), as well as their concentrations at different points of the cell cycle (reviewed in [3,26]).

We have previously shown that attenuation of p21 is anti-hypertrophic in renal mesangial cells in models of diabetic nephropathy[27], and plays an assembly factor role in vascular smooth muscle cells [20], but the function of this protein in RTEs has not been well investigated. Our present findings that ADPKD, which is characterized by increased RTE cell growth, may in fact be a disease which results from diminished p21 expression in affected tissues are consistent with recent data showing that polycystin-1 induces p21 [9] and causes resistance to apoptosis [23]. Our data is supported by another study demonstrating that immortalized proliferation of mouse embryonic fibroblasts from *PKD1*(-/-) mice occurs without the induction of p53 and (at late passage) p21 [28], as well a study showing that the CDK inhibitor roscovitine arrests disease progression[10].

There is a clear gender dimorphism with respect to progression in patients with ADPKD, with male patients progressing at a more rapid rate than female patients [13,14]. Consistent with this finding, our data indicate reduced expression of p21 in cystic kidneys from male, but not female, Han:SPRD rats. Estrogen has been shown to upregulate p21 in vitro[29], and may have contributed to the higher p21 levels in the cystic female rats in the present study. These observations are consistent with the hypothesis that suppression of p21 may play at least a permissive role in cystogenesis

p21 has been shown to have both assembly factor [19,20] and CDK inhibitory [4,17,18] functions, depending on the cell type examined. Thus, it was not clear at the outset that p21 would be cell cycle inhibitory or permissive in RTEs. Using an assay of S-phase cell cycle transit in a canine RTE cell line, we confirmed that the role of p21 in RTE cells is growth-suppressive. This data is supportive of our contention that the absence or diminution of p21 in cysts from PKD animal models may be pathogenic for cyst formation.

**Figure 8:**
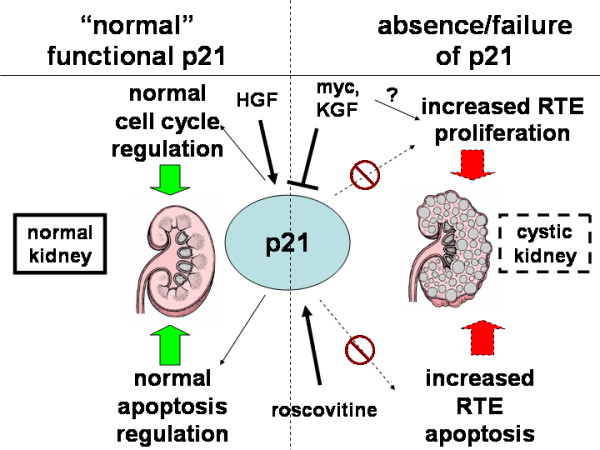
Hypothesis: attenuated or dysfunctional p21 contributes to cyst formation in ADPKD. It is likely that "normal" p21 prevents renal tubular epithelial cells (RTE) from assuming a proliferative, cystic configuration and thwarts the increased apoptosis that is seen with attenuated or maldistributed p21 which we hypothesize occurs in PKD. Myc and KGF contribute to cyst formation likely in part through p21 attenuation; roscovitine increase p21 leading to its salutary anti-proliferative effect in RTEs.

Roscovitine is a potent inhibitor of several cyclin-cdk pairs and is in clinical trials as an anti-cancer agent. Due to the similarity of PKD with neoplasia given the monoclonal origins of this disease, the recent demonstration of its long-lasting beneficial effect in a murine PKD model [10] was not unexpected. While investigation in non-cancer cell lines has not to our knowledge been reported, other investigators have shown a small decrease in p21 levels *in vitro *in prostate tumor spheroids [30]. Our finding of a dose-dependent increase in p21 with roscovitine in RTEs in the absence of increased apoptosis is consistent with our scheme for the function of p21 in PKD in these cells (Fig. 8), and, furthermore, suggests a mechanism for this newly identified therapeutic.

The mammalian target of rapamycin (mTOR) inhibitors, such as rapamycin, are now being recognized as having salutary effects in cyst formation and disease progression in human and animal models of ADPKD [31-33]. These compounds have recently been shown to be of benefit in kidney cancer [34], a disease with similarities to PKD, and p21 serves as a prognostic marker in renal cell carcinoma [35]. Furthermore RAD001, a rapamycin analog, has been shown to sensitize tumor cells to apoptosis through inhibition of p21 translation [36], which is consistent with this CKI being a potential target in a variety of malignancies [26] as well as in diabetic renal hypertrophy [27,37]. However, our finding that p21 is decreased in ADPKD, while in agreement with earlier studies showing that PKD1-deficient cells express lower p21, is not in complete concordance with the mTOR inhibitor data. This discrepancy may be explained by the pleiotropic effects of p21, as well as the stage at which p21 is inhibited: it is possible that a decrease in p21 levels in early stages of the disease allows RTEs to grow and form cysts via its cyclin/cdk inhibitor function, whereas mTOR inhibitors may be useful in later stages of the disease to decrease p21 and thereby attenuate cell growth via its assembly factor role [20]. When p21 is phosphorylated by upstream molecules such as phosphorylated Akt, p21 is translocated into the cytosol to act as an anti-apoptotic protein. Therefore, a decreased cytosolic p21 may result in inducing apoptosis which is the same effect as caspase inhibition [38]. It is also possible that, due to the global decrease in protein translation by mTOR inhibitors, p21 attenuation is unrelated to the effect of these drugs in PKD.

To further examine the mechanism by which cyst formation occurs by means of p21 attenuation, we utilized the growth factor HGF. MDCK cells spontaneously form cysts when cultured in 3D collagen gels and undergo *in vitro *tubulogenesis when treated with HGF; thus HGF is functions as a molecular "switch" from tubule formation to cyst [15]. We showed that p21 is in fact induced by HGF treatment in MDCK cells, consistent with data from other cell lines [39], and consistent with our hypothesis that p21 mediates the tubulogenic, not the cystic, phenotype.

Other than the genes identified as mutant in PKD (*Pkd1 *[the wild-type of which induces p21 as described above] and *Pkd2*), there is a striking paucity of gene targets in this common genetic disease. Since we have now shown that p21 possesses an anti-proliferative, and hence likely anti-cystic, effect on the cystogenic MDCK cell line, and that p21 is decreased in human ADPKD, it is possible that the p21 pathway, including the *p21*^*waf1 *^gene, may be utilized in future therapy of this disease with or without the addition of another CDK inhibitor such as roscovitine which itself increases p21. The finding by other investigators that inhibition of apoptosis, as would be expected to occur with increase levels of p21, slows ADPKD disease progression [38] is another potential benefit of such gene therapy. We foresee the ultimate use of CDK inhibition, either in embryonic or later life, as a possible PKD preventative measure.

## Conclusion

We have shown that p21, a cyclin kinase inhibitor which mediates cell cycle progression as well as apoptosis, is decreased in a rat model as well as in human ADPKD, and that roscovitine, which has been shown to have a salutary effect in an animal model of this disease, increases levels of p21. Validation of a potential pathogenetic model of increased cyst formation in ADPKD due to enhanced epithelial proliferation and apoptosis mediated by p21 suggests a mechanism for the salutary effect of roscovitine in this disease and supports further investigation of p21 as a target for future therapy of this disease.

## Competing interests

The author(s) declare that they have no competing interests.

## Authors' contributions

All authors have read and approved the final manuscript. JYP, WES, and JNL performed all experiments. TTO assisted in experimental design and execuation. SA and SPB facilitated human tissue procurement. SA helped with study design, conceived all of the rat experiments, and helped write the manuscript. RHW conceived of the study and wrote the original and final versions of the manuscript.

## Pre-publication history

The pre-publication history for this paper can be accessed here:


